# Acceptance and Willingness to Pay for Vaccine Against Human Papilloma Virus (HPV) Among Parents of Boys in Central Vietnam

**DOI:** 10.3389/fpubh.2022.801984

**Published:** 2022-03-10

**Authors:** Lan Hoang Nguyen, Thuy Bich Thi Le, Nhu Quynh Nguyen Le, Nhan Thanh Thi Tran

**Affiliations:** Faculty of Public Health, University of Medicine and Pharmacy, Hue University, Hue, Vietnam

**Keywords:** HPV vaccine, acceptance, parents, male adolescent, willingness to pay

## Abstract

Human papilloma virus (HPV) vaccine for adolescents was recommended as an effective prevention strategy of HPV-related cancers. In Vietnam, HPV vaccination has not been introduced to male adolescent. This study was conducted to examine the acceptance of having boys vaccinated against HPV and its underlying reasoning, and to identify their parent's willingness to pay (WTP) for HPV vaccination in central Vietnam. 785 parents of boys were directly interviewed based on a structured questionnaire. Parent's acceptability of HPV vaccine for their sons was identified by one question with response on 3-point scale (agree, don't know, and disagree). Multivariate logistic regression model was used to determine contributing factors to participant's acceptance. Bidding game method was applied to elicit WTP values for HPV vaccination with initial bid of 161.2 USD. The results showed that 49.2% of parents agreed to have their sons vaccinated against HPV. Factors that influenced parent's acceptance including son's age older than 12 years (*OR* = 1.5; 95% *CI*: 1.08–1.98); being eldest son (*OR* = 1.6; 95% *CI*: 1.13–2.19), being mother (*OR* = 1.4; 95% *CI*: 1.01–1.91), parents with high educational level (*OR* = 1.7; 95% *CI*: 1.11–2.47) and their knowledge of HPV and HPV vaccine (*OR* = 1.8; 95% *CI*: 1.23–2.65). Average WTP value for full doses of HPV vaccine was 137.5 USD, ranging between 9 USD and 188.3 USD. Parents' knowledge of HPV and HPV vaccine was the only factor affecting WTP value (Rho: 0.11; *p*-value: 0.030). The findings suggest a strategy be introduced for HPV vaccination to male adolescents in Vietnam.

## Introduction

Human papilloma virus (HPV) is a common group of viruses that are mainly transmitted through sexual contact. Globally, HPV infection has been considered as one of the most common viral infections with an estimation of 32.1% HPV-positive women in 2011. In men, the global prevalence rate of genital HPV infection is almost similar to that in women (2–44%). The prevalence of HPV infection is higher in developing countries and young people (1). There are more than 100 HPV types, and high-risk types are responsible for oropharyngeal cancers and anogenital cancers in both sexes such as cervical, anal, vulvar, vaginal, and penile cancers. Worldwide, cervical cancer is ranked fourth among most common cancers in women with an estimated 570,000 new cases in 2018 representing 7.5% of all female cancer deaths: more than 85% of these occurred in low- and middle-income countries (2, 3).

Human papilloma virus vaccine has been considered as an effective prevention strategy for HPV-related cancers. Until now there are three HPV vaccines, Cervarix for females, Gardasil and Gardasil-9 for females and males, which are licensed by the US Food and Drug Administration (FDA) (4). The Centers for Disease Control and Prevention (CDC) in the USA recommends HPV vaccination for adolescents aged 11–12 years, but it can start at age 9 through 26 if they were not adequately vaccinated previously. Two doses of HPV vaccine are recommended for adolescents ages 9–14 while three-dosed schedule is for people ages 15–26 (5).

According to WHO, 100 countries introduced HPV vaccine into their national vaccination program by 2019; however, they have focused on girls and young women as target population (6). Medical literature proved that HPV vaccination of boys has many benefits including preventing genital warts and HPV-related anogenital and oral cancers in men, preventing anal cancer in MSM group (Men who have Sex with Men) as well as preventing transmission of HPV to female sexual partners, which would decrease HPV-related anogenital cancers in women (7), but HPV vaccination for adolescent boys was approved in few countries and therein rates of vaccine coverage in this group have still much lower than in female group (8–11). Currently, a meta-analysis from 79 studies in 15 countries revealed that proportion of HPV vaccine uptake among parents for their daughters, sons, and both was 46.5, 20.3, and 39.8%, respectively (8). For the countries which offered HPV vaccination for boys, common reasons to impede HPV vaccine uptake among the parents were limited knowledge of HPV vaccine benefits for boys, awareness of availability of HPV vaccine for males, and out of pocket cost of the vaccine (12–16).

In Vietnam, the prevalence of HPV infection among women varied across population ranging between 2 and 11% (17). Information about HPV infection in men was still limited. However, current reports showed that prevalent rate of HPV infection was 25% among male patients with sexually transmitted infections (STIs) and was 23 to 79.6% among patients with penile cancer (18, 19). In 2008, the Ministry of Health approved two types of HPV vaccines including Cervarix and Gardasil with the purpose of preventing cervical cancer for girls and young women. Because these vaccines are not included in the expanded immunization program of the country, users must pay for HPV vaccination, ranging from 45 to 100 USD per dose depending on the provider. Consequently, coverage of HPV vaccination in target group in Vietnam was low. One of the main obstacles in expanding the HPV vaccination program was thought to be lack of national budget for the implementation. Presently, with the support of some international organizations, Vietnam would plan the expansion of HPV vaccination in the country in accordance with the priorities of the local health sector. Consequently, users only pay a much lower price than market price to receive HPV vaccine (20). However, target population for the expansion is still girls only. In line with recommendation of HPV vaccination for males as one of effective solutions for fighting cervical cancers in females as well as HPV-related cancers in both sexes (7), the present study was conducted with the aim of examining acceptance for HPV vaccination for boys and its deciding factors among their parents, and identifying willingness to pay for HPV vaccination for their son. Findings from the study would provide evidences to support Ministry of Health to introduce HPV vaccine to male adolescents in the country.

## Materials and Methods

### Study Setting and Participants

A cross-sectional study was conducted at secondary schools in Hue city which is the capital of Thua Thien Hue province located in central Vietnam. The city has 27 wards which are administrative divisions of the city. These wards are divided into two regions, north and south of Huong river. In 2020, the city has 24 secondary schools with 19,600 students. In Vietnam, secondary schools include 4 grades from sixth to ninth grade, which usually involve students ages 11–15.

The study randomly selected four schools as representatives of geographical regions of the city. Two schools are among 13 schools on the north and others are among 11 schools on the south of the river; then at each region, one school in the central city and another in its periphery were also randomly chosen on the basis of their location on the map of the city. Participants, who were parents of male students and agreed to enroll in the study, were invited to interview. In each school, we conducted interviews after class with the support of homeroom teachers. A total of 785 parents completed the interview giving a response rate of 92.6%.

### Data Collection

Data were collected in December 2020 when all schools organized a parent meeting at the beginning of a new school year. After the meeting finished, parents were interviewed directly at the classroom using a structured questionnaire. The interviewers, who were staff and graduated students of the Faculty of Public Health in University of Medicine and Pharmacy, Hue University, were trained on the content of the questionnaire and skills of interview.

### Instruments

#### Structured Questionnaire

The questionnaire consisted of three parts: part 1 was personal characteristics of participants and their sons, family history of HPV-related diseases and HPV vaccination of sons; part 2 was questions to assess parent's knowledge of HPV infection and HPV vaccine; and part 3 was about parent's acceptance and the price of WTP for HPV vaccination for their sons. The questionnaire was pre-tested on 20 random parents of boys ages 11–15 in Hue city and revised before implementing data collection. These parents were not selected from study settings.

#### Assessment of Knowledge About HPV Infection and HPV Vaccine

The knowledge about HPV infection and HPV vaccine was measured by 14 statements (true/false/don't know), with the convention that 1 point for a correct answer and 0 point for wrong or “don't know” answer. The maximum possible total score for knowledge was 14. The knowledge was rated as “good” for total score of 7 and above; total scores below 7 were characterized as “not good.” The questions about knowledge of HPV infection and its vaccine were developed on the basis of document on HPV of CDC (21). The reliability of the tool assessing knowledge was evaluated using Cronbach's alpha. The results showed that the internal consistency of the tool was good with Cronbach's alpha of 0.90.

#### Acceptance and Eliciting WTP

Parent's acceptability of HPV vaccine for their sons was identified by one question “do you agree to vaccinate against HPV for your son?” with response on 3-point scale (agree, don't know, disagree). Parents who chose the answer “agree” were continued to be interviewed to obtain a maximum price that they were willing to pay for full doses of HPV vaccine for their son. The bidding game method of contingent valuation was used to elicit WTP price for two doses of Gardasil vaccine. In this study, the initial bid was 3,580,000 VND (about 161.2 USD) that was the current market price for two doses of Gardasil at the time of the survey. The parents would be asked “are you willing to have your son vaccinated against HPV with a price of 3,580,000 VND?” If the answer was “yes,” the next questions would increase price incrementally by 200,000 VND (9 USD) until the answer was “no.” Then maximum WTP was final price that received the answer “yes.” If their answer was “no,” the next questions would decrease price incrementally by 200,000 VND until the answer was “yes,” and that was maximum WTP. These incremental levels were on the basis of pre-survey on 20 parents of boys ages 11–15 in Hue city. In the study, the lowest price was suggested to be 2,980,000 VND (134.2 USD); however, parents could choose any price in case they could not accept that lowest price. WTP price was presented in both VND and USD. The exchange rate used was that in effect on December 15, 2020 (1 USD = 22,203 VND).

### Data Analysis

Descriptive statistics including frequency and percentage was used to summarize the personal characteristics of parents and their sons and the acceptability of HPV vaccination for sons. Multivariate logistic regression model was employed to identify the determinants of parent's acceptability of HPV vaccination for their sons. WTP for HPV vaccination was presented by mean, *SD*, median, and min–max. However, data of WTP was not a normal distribution; non-parametric tests (Mann–Whitney *U* test was used to compare two groups and Kruskal–Wallis test was used to compare three groups) and spearman correlations were used to reveal factors affecting WTP price. An alpha value of 0.05 was considered statistically significant.

## Results

### Characteristics of Participants and Their Sons

Table 1 describes the main characteristics of 785 parents and their sons. Two-thirds of the participants were mothers. Mean age of parents was 42.9 (*SD* = 6.1) ranging between 29 and 62. Their educational levels were rather high with 44.8% of them attaining college degree or higher. More than half of the parents had unstable income. Twenty-one participants (2.7%) had no income; they were either unemployed or housewife. Most of the participants were maintaining marital status (94.6%). There were 24 parents (3.1%) whose households were classified as poor or near poor. Almost all of them (98.1%) reported that no one of their family suffered a HPV-related disease. The number of children of each parent ranged from 1 to 7 (mean 2.2; *SD* = 0.7) and their average number of sons were 1.5 (*SD* = 0.6) ranging between 1 and 5. Majority of the parents indicated that both father and mother were responsible for making decision on their children's health. The number of male students whose parents participated in the study was distributed higher into sixth and seventh grades. Their mean age was 12.4 years (*SD* = 1.2) ranging between 11 and 15. More than half of the boys were the eldest son. There were 22 sons (2.8%) who suffered either a chronic disease or a birth defect. None of boys was vaccinated against HPV before.

**Table 1:** Characteristics of participants and their sons (*n* = 785).

**Participants**		***n***	**Percentage**
Relations with students	Mother	472	60.1
	Father	313	39.9
Age (years)	Mean (SD): 42.9 (6.1)	Median: 42 Min: 29	Max: 62
Educational level	Secondary school and lower	249	31.8
	High school	184	23.4
	College and higher	352	44.8
Occupation	Stable income	349	44.4
	Unstable income	415	52.9
	No income	21	2.7
Marital status	Living with wife/husband	743	94.6
	Separation/divorce/widowed	42	5.4
Economic classification of household	Poor/near poor	24	3.1
	Normal	761	96.9
Family history of HPV related diseases	Yes	15	1.9
	No	770	98.1
Number of children	Mean (SD): 2.2 (0.7)	Median: 2 Min: 1	Max: 7
Number of sons	Mean (SD): 1.5 (0.6)	Median: 1 Min: 1	Max: 5
Decision-maker on children's health issues	Father	65	8.3
	Mother	199	25.3
	Both	521	66.4
**Their son**			
Grade	Sixth	207	26.4
	Seventh	226	28.8
	Eighth	165	21.0
	Ninth	187	23.8
Age (years)	Mean (SD): 12.4 (1.2)	Median: 12 Min: 11	Max: 15
The eldest son	Yes	416	53.0
	No	369	47.0
Chronic diseases/birth defect	Yes	22	2.8
	No	763	97.2
Have been vaccinated against HPV	Yes	0	0.0
	No	785	100

The knowledge about HPV and HPV vaccine of parents was described in Table 2. More than two-third of them had not ever heard of HPV. Only 18.9% of parents achieved good level of knowledge of HPV infection and HPV vaccine. Information including benefits of vaccine against HPV infection and HPV-related cancers, sex at risk due to HPV infection and who should be vaccinated against HPV were known by a larger percentage of the parents. In contrast, only a few of parents (<8%) knew ideal age to receive HPV vaccination, dose of HPV vaccine for children under 15 years old, and asymptomatic HPV infection.

**Table 2:** Knowledge about Human papilloma virus (HPV) and HPV vaccine of the parents (*n* = 785).

	***n***	**Percent**
**Heard of HPV**		
Yes	208	26.5
No/don't remember	577	73.5
**Knowledge about HPV and HPV vaccine**		
Good	148	18.9
Not good	637	81.1
**Right answers about HPV**		
HPV is a sexually transmitted disease	144	18.3
There are many types of HPV	130	16.6
HPV may infect both sexes	178	22.7
HPV can infect you without symptom	56	7.1
HPV infection can cause genital warts	116	14.8
HPV infection can cause cervical cancer in women	156	19.9
HPV infection can cause penis cancer in men	112	14.3
HPV infection can cause other anogenital and oropharyngeal cancers in both sexes	136	17.3
HPV infection can be prevented by vaccine against HPV	197	25.1
HPV-related cancers can be prevented by vaccine against HPV	198	25.2
Vaccine is used for people who are diagnosed free HPV infection	176	22.4
Both sexes are vaccinated to prevent HPV infection	191	24.3
The greater immune response to vaccinate against HPV is at ages 11 or 12 years	31	3.9
Children under 15 years old need two doses of HPV vaccine	58	7.4

### Acceptability of HPV Vaccine for Boys and Its Determinants

The study showed that 49.2% of the parents agreed to have their son vaccinated against HPV vaccine.

Results from multivariate logistic regression analysis revealed factors affecting parents' intention to have their sons vaccinated (Table 3). Parents of the boys who were older than 12 and were the eldest son were likely 1.5 times (*OR* = 1.5; 95% *CI*: 1.08–1.98) and 1.6 times (*OR* = 1.6; 95% *CI*: 1.13–2.19) to accept HPV vaccination for their sons more than their counterparts, respectively. The mothers were 1.4 times more likely than the fathers to agree to have their son HPV vaccinated (*OR* = 1.4; 95% *CI*: 1.01–1.91). The parents with good knowledge of HPV infection and HPV vaccine were more likely to accept HPV vaccine compared to those with worse knowledge (*OR* = 1.8; 95% *CI*: 1.23–2.65). The parents who attained college degree or higher were more likely to get HPV vaccine for their sons than those with a lower level of education (*OR* = 1.7; 95% *CI*: 1.11–2.47).

**Table 3:** Factors related to acceptability of parents for HPV vaccination for their sons^*^.

**Factors**		**OR**	**95% CI**	***p*-value**
Age of son	≤ 12	1		
	>12	1.5	1.08–1.98	0.014
The eldest son	No	1		
	Yes	1.6	1.13–2.19	0.007
Relations with son	Father	1		
	Mother	1.4	1.01–1.91	0.046
Educational level	Secondary school and below	1		
	High school	1.3	0.89–2.03	0.149
	College and higher	1.7	1.11–2.47	0.013
Knowledge of HPV and HPV vaccine	Not good	1		
	Good	1.8	1.23–2.65	0.003

* *This table presented only results which showed statistically significant associations*.

The study did not find a significant association between participant's acceptance of HPV vaccination for their sons and other variables including demographical characteristics of parents, their marital status and occupation, economic situation of household, family history of HPV-related diseases, number of children, number of sons, and disease of sons (*p*-values > 0.05) (Supplementary Table 1).

### Willingness to Pay for HPV Vaccination for Sons

On average, WTP amount for two doses of HPV vaccine was 3,053,005 VND (about 137.5 USD) ranging from 200,000 VND (9 USD) to 4,180,000 VND (188.3 USD). Among the parents who accepted to get their sons vaccinated, 63.7% of them were willing to pay <3,580,000 VND (161.2 USD) for two doses of Gardasil (Table 4).

**Table 4:** Willingness to pay for HPV vaccination for sons among the parents (*n* = 386).

		**VND**	**USD**
	Mean (SD)	3,053,005 (763,645)	137.5 (34.4)
**WTP**	Median	3,180,000	143.2
	Range	200,000–4,180,000	9–188.3
**Number of participants at WTP levels (cut off point = market price of Gardasil vaccine)**			
Cut off point = 3,580,000 VND ($162 US)		*n*	Percentage
	< cut off point	246	63.7%
	≥cut off point	140	36.3%

Table 5 showed that the knowledge of HPV and HPV vaccine was the only factor associated with WTP price for HPV vaccination among parents. This is a positive but weak correlation (Rho: 0.11). No other variables were found significantly related to WTP price for HPV vaccination in the study (*p* > 0.05) (Supplementary Tables 2A,B).

**Table 5:** Factors associated with WTP for HPV vaccine among the participants (*n* = 386).

		**WTP**
Total mean score of knowledge	Rho	0.11
	*p*-value	0.028

## Discussion

### Acceptability of HPV Vaccine for Boys

One noticeable finding is that no participant reported prior HPV vaccination for their sons (Table 1). In line with the recommendation of WHO, Vietnam introduced HPV vaccine for girls ages 9–13 as a public health measure against cervical cancer (20, 22). Accordingly, communication messages encouraging HPV vaccination have focused on adolescent girls. A study by Tran et al. (23) in Hanoi reported that 100% of the male participants thought HPV vaccine was for females only. Authors also indicated that women were more interested in information of HPV vaccine than men. In our study, although two-thirds of the parents were mothers, only 26.5% of them had ever heard of HPV or HPV vaccine (Table 2). This result was much lower than that those in previous studies in Vietnam and in the world (24–27). Nguyen et al. (24) found that 71.3% of women ages 18–49 in Hanoi were aware of HPV vaccine. A study in Thailand reported that 52% of the parents had heard of HPV and HPV vaccine (25). Sitaresmi et al. (26) showed that 49.2 and 48.8% of the parents in Indonesia had known about HPV infection and HPV vaccine, respectively, and there were 70.5% of the parents in Texas, USA who knew these issues (27). As a result, our participants had lack knowledge of HPV and its vaccine. Only 18.9% of the parents achieved good level of knowledge about HPV and HPV vaccine. Questions of benefits of vaccine against HPV received the highest percentage of correct answers while only a few of the parents knew correctly the ideal age for receiving vaccine and the recommended number of doses of vaccine for adolescents under 15 years old (Table 2). The results were similar to those of Nguyen et al. (24) among child-bearing aged women in Hanoi. Communication efforts should be improved to provide comprehensive information about HPV and its vaccine, especially including male adolescents as part of the target group. Not only does HPV vaccination for male protect them from getting HPV-related cancer, but also prevent the transmission of HPV to female sexual partners. This is necessary in light of the low coverage of HPV vaccination among girls and gender imbalance in Vietnam (28, 29).

Although the level of awareness of HPV and HPV vaccine was low among parents in the study, nearly half of them agreed to have their sons vaccinated against HPV in the future. The results from the multivariate analyses indicated that the strongest factor for accepting HPV vaccination was knowledge of HPV and HPV vaccine among the parents.

This finding is consistent with many previous studies in Vietnam as well as in other countries (20, 25, 31). A study in Thailand found that parents with better knowledge were related to higher acceptance of HPV vaccination for their children (25). A systematic review by Rodriguez et al. (30) showed similar findings. In Vietnam, Nguyen Minh et al. (31) demonstrated that a health education program on HPV knowledge improved the vaccination intention among mothers of teenage boys. Experiences from many countries also confirmed that in order to accept a vaccine, the population must have background knowledge about the importance of the vaccine and its safety (32, 33). Understandably, highly educated parents should have had more access to information sources regarding HPV and HPV vaccine and thus they were more willing to get their sons vaccinated against HPV than those with lower educational level. Our finding agreed with a study by Kimberly et al. (9) in the USA. However, in contrast to higher acceptation of the fathers toward vaccination for their children described in that study, we found that mothers expressed more desire to have their son HPV- vaccinated. This is explained by the difference in culture. In Vietnamese traditional society, mothers are expected to take good care of their children; they have stronger willingness to protect their children from diseases through vaccination. The finding is supported by a study in China which has the same familial culture (34). The Vietnamese traditional culture also explains for more willingness to get HPV vaccination for eldest son among the participants. According to this viewpoint, father and eldest son are decision-makers and spokespersons in a family (35). Son's age was also a factor in the parent's decision in the present study. The parents of boys older than 12 expressed more acceptances to have their son vaccinated than those of younger ones. This is in line with earlier studies. The parents thought that vaccination would affect a child's physical development (36) or believed that their children were not at risk because of no sexual activity (10, 13, 36, 37). The perception of the parents could lead to a missed opportunity to prevent their children from HPV infection. Indeed, a survey in Vietnam in 2009 found that 44% of adolescents age 14 or older had pre- marital sex, and the majority of them had unsafe first intercourse (38). Communication should emphasize the need of getting the vaccine before the first sexual experience and clearly indicate that a greater immune response can be achieved when vaccination happens at age 11 or 12 (5, 21).

### WTP for HPV Vaccination

The present study showed that there was a demand to get the sons vaccinated against HPV among their parents. Average WTP price was 137.5 USD for full doses of HPV vaccine, accounting for 6% of annual income per capita in 2020 in Vietnam (39). This value is 23.7 USD lower than current market price of two doses of Gardasil vaccine. Recently, Tran et al. reported that adult men were willing to pay an average amount of 166.2 USD for three-dosed course of HPV vaccine, accounting for nearly 7% of annual income per capita in Vietnam in 2016 (16). It is suggested that two doses of HPV vaccine for adolescents not only provide the most effective protection, but also save money for households. Average elicited WTP price was higher than that from earlier studies done in the world in terms of absolute monetary values as well as compared to annual income per capita. The average WTP for full dose of HPV vaccine was 8.85 USD in Ethiopia (40), 11.88 USD in Nigeria (41), 5.26 USD in Malaysia (42), a range of 9.23 USD to 30.78 USD in Thailand (43), and 252.71 USD in Chile (44). Apparently, the success in National Expanded Immunization Program in Vietnam has persuaded parents to believe in vaccine programs as a worthwhile investment for their children's health. Although the participants expressed a desire to get HPV vaccine for their son, two-thirds of the respondents were willing to pay the amount of money that was lower than the market price. The aforementioned studies also confirmed that cost of vaccination was a barrier to HPV vaccine uptake (30, 37, 45, 46). The Government's subsidies should be considered when it introduces HPV vaccine for male adolescents in the country.

The knowledge of parents about HPV and its vaccine were the only factor affecting WTP price from the study; however, this correlation was weak. A better awareness of HPV and HPV vaccine by the participants raised their WTP price for HPV vaccination. Similarly, studies found that individuals who recognized high risk of COVID-19 tended to pay more for vaccine than those who saw it as low risk (47, 48). Cerda et al. (49) also emphasized the importance of providing knowledge about COVID-19 to people to combat the pandemic.

The study has some limitations. First, this is a school-based survey; the sample was randomly selected but based on a list of parents who were present at the parent meeting of the school. Selection bias could be a potential problem. Second, the study was conducted in Hue city where many of traditional family values remained unchanged. In addition, income per capita in study setting was much lower than that in big cities of Vietnam. These can limit ability to generalize findings of the study. Third, the incremental level by 200,000 VND that resulted from a pilot survey could limit choice of respondents. Despite these limitations, this is the first study in Vietnam to identify the demand of HPV vaccine for male adolescents among their parents. The results can support health policy makers to introduce HPV vaccination for males in the country.

## Conclusion

There was a demand for HPV vaccination for male adolescents among our sampled parents; however, their WTP price for the current vaccine was lower than its market price. Public awareness of HPV and HPV vaccine for both sexes need to be strengthened and sustained. The vaccine against HPV should be introduced to male adolescents besides girls and young women in Vietnam. Government's subsidies should be considered to encourage HPV vaccination for both sexes in the country. This will contribute to reduce not only HPV-related diseases in men, but also the risk of cervical cancer in women.

## Data Availability Statement

The raw data supporting the conclusions of this article will be made available by the authors, without undue reservation.

## Ethics Statement

The study proposal was approved by the Ethics Committee for Biomedical Research of University of Medicine & Pharmacy, Hue University (No: H2020/082, dated June 3, 2020). In addition, approval for data collection at the sites was obtained by directors of secondary schools. The interview of study subjects was performed with their verbal permission after they were given adequate information about the study.

## Author Contributions

LN: conception, design of the study, data analysis, and writing the manuscript. TL and NL: data collection, data entering, and data analysis. NT: data collection. All authors have read and agreed to the published version of the manuscript.

## Funding

The research was supported by the research funds from Hue University (DHH 2020- 04- 113).

## Conflict of Interest

The authors declare that the research was conducted in the absence of any commercial or financial relationships that could be construed as a potential conflict of interest.

## Publisher's Note

All claims expressed in this article are solely those of the authors and do not necessarily represent those of their affiliated organizations, or those of the publisher, the editors and the reviewers. Any product that may be evaluated in this article, or claim that may be made by its manufacturer, is not guaranteed or endorsed by the publisher.
