# Supplementary material for: Acceptance and Willingness to Pay for Vaccine Against Human Papilloma Virus (HPV) Among Parents of Boys in Central Vietnam
Source: Front Public Health. 2022 Mar 10;10:801984. doi: 10.3389/fpubh.2022.801984 (PMC8960026; doi:10.3389/fpubh.2022.801984)
Supplement: Supplementary file 1 [file Table_1.pdf]

Table 1. Results of multivariate logistic regression model identified factors related to acceptability of parents for HPV vaccination for their son

**Factors**		**OR**	**95% CI**	**p- value**
Age of son	≤ 12	1		
	> 12	1.46	1.08 –1.98	0.014
The eldest son	No	1		
	Yes	1.57	1.13 – 2.19	0.007
Defect/chronic disease of son	Yes	1		
	No	0.82	0.33 – 2.01	0.664
Relations with son	Father	1		
	Mother	1.38	1.01 – 1.92	0.045
Age of parents	> 40 years old	1		
	≤ 40 years old	1.03	0.73 – 1.45	0.863
Educational level	Secondary school and below	1		
	High school	1.35	0.89 – 2.03	0.150
	University and higher	1.65	1.11 – 2.46	0.014
Residence	Hue city	1		
	Other	0.26	0.06 – 1.11	0.069
Marital status	Living with wife/husband	1		
	Separation/divorce/widow	1.74	0.87 – 3.45	0.115
Characteristics of occupation	No income	1		
	Stable income	1.78	0.67 – 4.69	0.245
	Unstable income	1.46	0.57 – 3.72	0.434
Economic situation	Poor/near poor	1		
	Non poor/near poor	1.36	0.56 – 3.32	0.495
Number of children	≥ 3	1		
	≤ 2	0.99	0.69 – 1.43	0.972
Number of son	≥ 2	1		
	1	0.97	0.72 – 1.31	0.839
Family history of HPV-related cancers	Yes	1		
	No/don’t remember	1.09	0.37 – 3.22	0.872
Decision maker for health problem of their children	Both	1		
	Father	1.38	0.80 – 2.39	0.245
	Mother	0.75	0.53 – 1.08	0.124
Knowledge of HPV and HPV vaccine	Not good	1		
	Good	1.81	1.22 – 2.67	0.003

Table 2A. Results of Spearman correlation to identify factors associated with WTP for HPV vaccine among participants

**Factors**	**WTP**	
	**Rho**	**p- value**
Total mean score of knowledge	0.11	0.028
Parent’s age	0.05	0.288
Son’s age	-0.05	0.352
Number of children	-0.08	0.130
Number of sons	-0.06	0.240

Table 2B. Results of non-parametric tests to identify factors associated with WTP for HPV vaccine among participants (n=386)

**Factors**		**n**	**Median (VND)**	**Range (VND)**	**p- value**
Relation with male student	Mother	244	2,980,000	3,980,000	0.159
	Father	142	3,180,000	3,880,000	
Residence	Hue city	383	3,180,000	3,980,000	0.410
	Other	3	3,580,000	600,000	
Educational level	Secondary school and below	96	2,980,000	3,880,000	0.148
	High school	87	2,980,000	3,480,000	
	University and higher	203	3,180,000	3,980,000	
Characteristics of occupation	No income	8	2,980,000	3,780,000	0.455
	Stable income	195	3,180,000	3,980,000	
	Unstable income	183	2,980,000	3,880,000	
Economic situation	Non poor/near poor	377	3,180,000	3,980,000	0.970
	Poor/near poor	9	3,180,000	600,000	
Marital status	Living with wife/husband	362	3,180,000	3,980,000	0.343
	Separation/divorce/widow	24	2,980,000	3,680,000	
Family history of HPV-related cancers	Yes	8	3,080,000	600,000	0.935
	No/don’t remember	378	3,180,000	3,980,000	
Decision maker for health problem of children	Father	35	3,380,000	3,680,000	0.530
	Mother	90	2,980,000	3,980,000	
	Both	261	3,180,000	3,880,000	
The eldest son	No	155	3,180,000	3,780,000	0.520
	Yes	231	2,980,000	3,980,000	
Defect/chronic disease of son	No	375	3,180,000	3,980,000	0.137
	Yes	11	2,980,000	3,180,000	

Mann-Whitney U test was used to compare two groups

Kruskal- Wallis test was used to compare three groups
